# Heritage‐specific trends in willingness, motivators, and barriers to Alzheimer's disease trial participation among Hispanic/Latino and Non‐Hispanic White adults

**DOI:** 10.1002/alz70860_106946

**Published:** 2025-12-23

**Authors:** Christian R Salazar, Rachel Kinder, Michelle M Nuño, Mary Ryan Baumann, Maricela Cruz, Jane E Manweiler, James Dayton, Josh D Grill

**Affiliations:** ^1^ Institute for Memory Impairments and Neurological Disorders, University of California, Irvine, Irvine, CA, USA; ^2^ ICF, Burlington, VT, USA; ^3^ University of Southern California, Los Angeles, CA, USA; ^4^ University of Wisconsin‐Madison, Madison, WI, USA; ^5^ Kaiser Permanente Washington Health Research Institute, Seattle, WA, USA; ^6^ University of California, Irvine, Irvine, CA, USA; ^7^ The UC Irvine Institute for Memory Impairments and Neurological Disorders, Irvine, CA, USA

## Abstract

**Background:**

Latinos enroll in Alzheimer's disease (AD) clinical trials at lower rates, yet it remains unclear whether their willingness differs from their non‐Hispanic White (NHW) counterparts. This study examined differences in trial willingness, along with barriers and motivators, among Latino and NHW adults in a nationally representative sample.

**Method:**

A structured web panel survey was administered to 1,800 adults aged 50 and older with no self‐reported neurological disorders, including 600 NHW and 1,200 Latino participants across the U.S. Participants received a primer on AD and clinical trials before responding. Willingness to participate was assessed using Likert‐scale items on general and AD‐specific trial participation. Multinomial logistic regression examined willingness differences, adjusting for age, sex, education, income, and AD family history. Secondary analyses explored motivators and barriers.

**Result:**

Among Latinos, 34.6% identified as Puerto Rican, 26.9% as Mexican, 14.4% as Central or South American, 8.5% as Cuban, 3.7% as Dominican, and 11.9% as other/mixed heritage. While overall trial willingness did not differ between NHW and Latinos, a trend in heritage‐specific differences emerged. Cuban (OR=2.30; 95% CI: 0.73–7.28) and Central American (OR=1.89; 95%CI: 0.45–7.93) participants showed a pattern of greater willingness compared to NHW, while Dominican (OR=0.60; 95% CI: 0.14–2.47) and Mexican participants (OR=0.78; 95%CI: 0.33–1.82) showed a trend toward lower willingness. Motivators also varied by heritage: Cubans prioritized health benefits (76.5%), research contributions (55.1%), and monetary incentives (40.9%), while Central Americans valued health benefits (68.2%), monetary incentives (50.6%), and physician recommendations (34.7%). Barriers also differed: Dominicans cited safety concerns (48.2%), visit burden (27.8%), and transportation (24.2%), while Mexicans noted visit burden (31.3%), safety concerns (29.3%), and time constraints (20.8%). More Latinos reported they would be “more willing” to participate in AD‐focused trials than NHW (42.6% vs. 36%, *p* = 0.0069).

**Conclusion:**

Willingness is not universally a barrier for Latinos, but heritage‐specific trends underscore the need for culturally tailored recruitment strategies. Addressing logistical barriers, safety concerns, and emphasizing trust and societal benefits is key to improving Latino participation in AD trials.

